# A moment for compassion: emerging rhetorics in end-of-life care

**DOI:** 10.1136/medhum-2017-011329

**Published:** 2018-02-10

**Authors:** Shahaduz Zaman, Alexander Whitelaw, Naomi Richards, Hamilton Inbadas, David Clark

**Affiliations:** 1 School of Interdisciplinary Studies, University of Glasgow, Dumfries, UK; 2 Department of Global Health and Infection, Brighton Sussex Medical School, University of Sussex, Brighton, UK

**Keywords:** end of life care, palliative care, care of the elderly, Public Health, medical anthropology

## Abstract

Compassion is an emotional response to the suffering of others. Once felt, it entails subsequent action to ameliorate their suffering. Recently, ‘compassion’ has become the flagship concept to be fostered in the delivery of end-of-life care, and a rallying call for social action and public health intervention. In this paper, we examine the emerging rhetorics of compassion as they relate to end-of-life care and offer a critique of the expanding discourse around it. We argue that, even where individuals ‘possess’ compassion or are ‘trained’ in it, there are difficulties for compassion to flow freely, particularly within Western society. This relates to specific sociopolitical structural factors that include the sense of privacy and individualism in modern industrialised countries, highly professionalised closed health systems, anxiety about litigation on health and safety grounds, and a context of suspicion and mistrust within the global political scenario. We must then ask ourselves whether compassion can be created intentionally, without paying attention to the structural aspects of society. One consequence of globalisation is that countries in the global South are rapidly trying to embrace the features of modernity adopted by the global North. We argue that unrealistic assumptions have been made about the role of compassion in end-of-life care and these idealist aspirations must be tempered by a more structural assessment of potential. Compassion that is not tied to to realistic action runs the risk of becoming empty rhetoric.

## Introduction

Compassion is hard to argue against. It is an emotional response to the suffering of others ‘when we either see it, or are made to conceive it’.^1^ But compassion is not merely a passive sense of pity, it is also about engagement—seeking to assist those whose suffering may be ameliorated by our actions. Compassion, therefore, has emotional, social and practical dimensions. In the words of Lauren Berlant, it is ‘an emotion in operation’.^2^ Its meaning and definition have been framed by theology, religious practice, psychology and even political theory. Compassion goes to the heart of what it is to be human and to exist in relationships, caring for our own welfare and also that of other people. Currently, we observe compassion being fostered in many contemporary settings—from schools and police departments to business organisations and in the work of non-profit organisations. It can be found as a call to action, a disposition to be fostered or an ethic to guide strategy.

Compassion has come to occupy a particular place within the caring disciplines. It is recognised as a critical aspect of the art of nursing and medicine, and of effective communication, and is often considered essential if patient and family needs are to be met and the desired clinical outcomes are to be achieved. Today, it sits at the centre of many debates about how health and social care should be delivered.

In this context, ‘compassion’ has also been invoked by a variety of protagonists in the field of end-of-life care. So much so that it has become the flagship concept to support some end-of-life organisations and projects, or an attribute to be nurtured in the delivery of end-of-life care, a rallying call for community action and public health intervention. Convinced that compassion is ‘having a moment’ in contemporary end-of-life discussions, in this paper we examine and offer a critique of the emerging rhetorics of compassion in care of the dying and identify some of the key structural barriers to ‘freely flowing’ compassion, particularly within Western society.

## Concepts of compassion

‘Compassion’ occupies a central place in religious thought.^3^ Cherishing other living beings, the Buddhist perspective presents compassion as the mind that motivates one to release others from their suffering.^4^ In Hindu philosophy, compassion is the source of *dharma*, the right action and the basis of *ahimsa*, non-violence.^5^ Similarly, compassion represents the spirit of Islam, a central attribute that makes no distinction between the sufferings of others and that of one’s self.^6^ Beyond being a personal virtue, Christian theology articulates a social vision grounded in the understanding that God is compassionate.^7^ Compassion is related to concepts like ‘empathy’, ‘kindness’, ‘benevolence’ and ‘humanity’ but differs from them in that it necessarily includes an action component, which these other sentiments may not.

The role played by compassion in defining the ethical life of an individual or a community has also been of interest to anthropologists.^8^ Here, compassion is viewed as an emotion that is both configured by and also potentially configures a person’s moral and ethical assumptions.^9^ Anthropological interest in compassion has also extended to a discourse analysis of the ‘politics of compassion’, and the ways in which feeling or showing compassion can entail hierarchical power relations, linked to ideas of charity and condescension.^10^ Expressions of compassion can create a distance between a suffering and passive other, and a compassionate and active self.^11^ For example, Didier Fassin describes the ‘humanitarian reduction of the victim’, critiquing ways in which governments, the media and humanitarian organisations themselves attempt to summon compassion from the public or resist/deny compassion through judgements about the relative value of people’s lives—creating a ‘complex ontology of inequality’.^12^ He argues that compassion can reduce engagement with people’s suffering to the level of individual relations, emotions or affect, obscuring the root cause of suffering within the political system or global injustice.

Compassion has also been discussed widely in more applied contexts, particularly within the care literature. Here, it is considered the foundational emotion for developing a caring disposition and for identifying who have care needs. Some complications are however recognised as being associated with this articulation. Conceptually, the relationship between practical compassionate care and the wider religious and ethical bases of compassion is ambiguous. Watkins,^13^ for example, suggests that the ‘religious connotations’ of compassion do not ‘sit comfortably in the lexicon of contemporary healthcare professionals’ and seem to ‘elevate the practice of caring to a vocational height too lofty for modern practitioners’, one that ‘grates against the professional dispassionate stance’.^13^

More pragmatically, within a ‘production line’ approach to health services, a perception exists that compassion may have been squeezed out of modern, routinised care, resulting in a form of ‘institutionalised heartlessness’.^14^ Similar structural constraints can also result in carers experiencing states that lead to a defensive response to suffering and what is known as compassion fatigue.^15^ This includes ‘secondary trauma’, an emotional reaction that arises from experiencing the distress of another, and ‘moral distress’, a dissonance that is created when institutional constraints limit what carers believe to be truly compassionate care.^16 17^ Furthermore, we note the existence of a series of scandals around poor or even criminal care, where compassion has been notable by its absence.^18^

## Harnessing compassion to end-of-life care

‘Compassion’ does not seem to have been a term widely used by the hospice and palliative care movement in the 1960s and 1970s and does not appear at all, for example, in the selected writings of Cicely Saunders.^19^ But from the 1990s, we see a flurry of interest in compassion relating to end-of-life issues in a number of areas, where compassion is considered to be a component in facilitating a ‘good death’.

First, there are examples where the notion of compassion is invoked within the name of a particular organisation. Here, compassion is associated with choices, rights, and a fair and balanced set of options for the healthcare ‘consumer’, even at the end of life. Thus, *Compassion in Dying* is the UK charity that seeks to inform and empower people to exercise their rights and choices relating to end-of-life care and to plan ahead to ensure their wishes are respected. It is also the sister charity to another which campaigns for the legalisation of assisted dying.^20^ Similarly, *Compassion and Choices* in the USA is the largest non-profit organisation working towards the legalisation of assisted dying.^21^ Attempting to position assisted dying within the rhetorics of compassion has not gone unchallenged by those who oppose legalisation. Pope Francis himself declared that assisted dying offers ‘false compassion’^22^ and many palliative care organisations adopt the theme of compassion to describe what they do. This is reminiscent of the dispute between pro-assisted dying and anti-assisted dying campaigners over the term ‘dignity’. When the UK’s Voluntary Euthanasia Society applied to change its name to Dignity in Dying in 2006, The Association of Palliative Medicine spearheaded the opposition, describing it as a ‘dishonest’ attempt to seek a ‘monopoly’ over a term that also meant receiving good palliative care.^23^ It appears that both ‘dignity’ and ‘compassion’ are terms used by different groups in the end-of-life ‘space’, and sometimes their goals and ambitions are deeply oppositional in character.

Second, we see a turn to compassion in aspects of clinical care at the end of life. But findings from one scoping review of the wider healthcare literature show that compassion is rarely studied empirically.^24^ On the basis of in-depth narrative interviews with leaders in the field of palliative care, a recent monograph concludes with a set of questions about what it means to be a compassionate practitioner of end-of-life care, describing how compassion is demonstrated in practice and how it can be fostered and taught.^25^

Third, and perhaps most significantly, compassion has become the orienting principle for ‘new public health’ approaches to end-of-life care, first developed in the notion of ‘compassionate cities’ by Allan Kellehear. This approach is rooted in a health-promoting orientation to end-of-life care.^26^ Its starting point is that end-of-life care is ‘everybody’s business’ and must not be abandoned to the health and social care system. The phrases ‘compassionate cities’ and ‘compassionate communities’ convey ideas about reclaiming death and bringing it back to its appropriate setting—the home and community—as well as regaining ‘lost’ knowledge about the dying process. This approach seeks to nurture compassionate communities and adopt an asset-based and social capital orientation to palliative care. There are now many initiatives around the world establishing a ‘compassionate communities’ dimension to end-of-life care. Two ways of doing this have been identified.^27^ One is to develop compassionate communities in a wider sense and then make their resources available to those at the end of life. Another is to use end-of-life care situations in ways that allow people to access local networks and thereby help to develop a compassionate community. The concept is found at the local level in the global north, for example in ‘Compassionate Inverclyde’, Scotland, the Milford Care Centre ‘Compassionate Communities Project’, Ireland, and the ‘Compassionate Community Network’, Australia. There are also initiatives in the global south, such as ‘Compassionate Kodjikode’, in India, and ‘Compassionate Korail’, Bangladesh.

The sources of compassionate action and perceptions of how it can be promoted are, however, a matter of contention. Some see compassion as an ‘art’ with its own ‘value’ or ‘virtue’,^14 28^ while others see compassion in more tangible and functional terms, for example in the notion of a knowledge-based ‘science’ of compassionate caring.^29^ The latter suggests that compassion is something that can be taught or actively fostered via, for example, staff training, care modelling or providing resources to reduce ‘compassion fatigue’.^30^

We argue that even where individuals ‘possess’ compassion or are ‘trained’ in its components, it is nevertheless difficult for compassion to flourish, whether in clinical settings or in communities. Within Western post-industrial societies, this is seen most starkly in a set of structural barriers to what we term ‘free flowing’ compassion—that is compassion ‘in operation’ as a form of practical action, observable in settings across the whole of a society.

## Structural barriers to free-flowing compassion

The sense of privacy and individualism in Western culture is one barrier to the practice of free-flowing compassion. Although notions of privacy exist in any culture, we suggest that these take a heightened form in Western post-industrial societies and will therefore inhibit the development of ‘compassionate communities’ for end-of-life care. Kate Fox, while writing about English life, jokingly observes that “the privacy rules are so obvious that you could spot them from a helicopter, without even setting foot in the country”.^31^ This heightened form of privacy is also a result of the high value placed on individualism in industrialised Western society. Individualism advocates independence and self-reliance over group values. Geert Hofstede in his work on ‘comparative cultural dimensions’ discussed how certain societies nurture ‘I’ consciousness rather than ‘we’ consciousness.^32^ Robert D Putnam famously suggested that community has been lost in American society, where people now ‘bowl alone’, with a resultant decline in the ability to interact and in social capital.^33^ In the end-of-life context, this can create tensions for people who have always valued their independence and self-reliance and now see these compromised as a result of bodily decline and care needs that can be met only through dependence on others. Matt Dawson discusses how in the post-industrial states individuals are left to create their own identity free from collective definitions and categorisations while at the same time they experience a greater sense of individual responsibility due to the privatisation of previously collective decisions.^34^

Public and professional behaviour in Western countries is shaped by strict legal and regulatory codes.^35^ These promote the thinking that dying requires professionals because they will know how to conform to regulatory health and safety requirements. Knowing that such professional and organisational codes exist influences and constrains public behaviour. The fear of inadvertently transgressing causes a degree of public anxiety and reluctance for the lay person to get involved in end-of-life care. However, in order for compassion to flow freely, free access is required to those in need. Now the anxiety of litigation on health and safety grounds is a major barrier for free-flowing compassion within Western society. A scanning exercise conducted by the Dying Matters Coalition for *Compassionate Communities in England* states that “genuinely held fears for the safety of the cared-for person were felt by many, when working with the community to provide support. Fears about vulnerable adult protection, risk management, and confidentiality were all raised”.^36^ One frustrated volunteer in the scanning exercise stated:

…We’ve got healthcare so wrapped up in risk aversion and NHS bureaucracy that nurses were saying ‘I’m not sure about this and that’, but for goodness sake, people have gone to have a cup of tea in people’s houses for centuries.

Hofstede also described societies according to the degree of ‘uncertainty avoidance’ people display.^32^ In a society of ‘weak’ avoidance, the uncertainty inherent in life is accepted and there is a level of accommodation to ambiguity accompanied by a general dislike of rules. In a ‘strong’ avoidance society, the uncertainty inherent in life is felt as a continuous threat that must be fought, ambiguity is not tolerated and there is a need for clarity and structure as well as an emotional desire for rules. It is clear that the strong uncertainty avoidance of late modern industrialised societies has generated a cultural preoccupation with health and safety. It may well be a disposition that proves inimical to free-flowing compassion. Westerners live in a time that is heavily inflected by suspicion and the mistrust of others, with visible barriers to ‘neighbourliness’.^27 37^ The sense of suspicion has been significantly amplified in the wake of global terrorist attacks, particularly in Western cities. Likewise, ethnic and racial tensions have heightened in the contexts of immigration and the refugee crisis. Free-flowing compassion may struggle to assert itself in an atmosphere of apprehension and fear. Community involvement at the end of life means allowing an outsider into one’s home, and this may be a very difficult challenge to overcome.

Finally, the highly institutionalised and professionalised health systems in Western countries function as closed systems with very limited scope for lay participation and engagement.^26^ Public involvement has been seen by some as mere tokenism.^38^ Suresh Kumar is a leading proponent of community action in palliative care. He observes:

A system that does not have the room to use the various capabilities of lay people is an ‘underdeveloped’ system. This is why I would call the health systems in many European countries ‘underdeveloped’… (Kumar S, Unpublished, 2015).

Because there are only limited openings in Western health systems to incorporate lay participation, we contend that it will be difficult to integrate free-flowing compassion within them. It will likewise be hard to make death ‘everyone’s business’ or to regain ‘lost’ lay knowledge about the dying process in the developed Western context.

We maintain therefore that there are significant barriers at the structural level of post-industrial Western society which hinder the expression and practice of spontaneous compassion or allow it to flourish within formal systems of health and social care. We must then ask whether compassion can be intentionally evoked, without paying attention to these structural barriers.

## Conclusion

Compassion is undoubtedly ‘having a moment’ in contemporary end-of-life discussions. However, we suggest the need for caution if unrealistic expectations about it are to be avoided. In this context, might the global South, despite its barriers to healthcare access, have more potential to promote and practise free-flowing compassion among community members? ‘Reverse innovation’, defined as the flow of ideas from low to high income settings, has gained traction in the business world and only recently has been applied to the healthcare setting.^39^ Is there some potential to reverse the conventional relationship of ‘passive receiver of compassion’ (global south) *versus* ‘active giver of compassion’ (global North)? Although attempts have been made in the global North,^40 41^ perhaps the most refined version of a compassionate community for end-of-life care is found in the Neighbourhood Networks in Palliative Care, first developed in Kerala, India.^27 42^ This model has also inspired other community-based palliative care projects in the global South.^43^ One aspect of the success of the Kerala model lies in the fact that many of the structural obstacles present in Western post-industrial societies are absent.^44^

However, we must also note that, given strong globalising trends, countries in the global South are also rapidly trying to embrace the features of modernity adopted by the global North. The post-colonial thinker Dipesh Chakrabarty suggests that in this context Europe alone is seen to be fully modern, while the rest of the world retains many pre-modern elements and must reside in what he calls the ‘imaginary waiting room of history.’^45^ In this schema, the mission of the rest of the world is to try to catch up with Europe or the West. The recent ‘quality of death’ ranking by *The Economist* is a remarkable case in point.^46^ The ranking shows mostly the Western developed countries, following the conventional specialist palliative care model rather than the community development model, with the UK ranked highest. There is an implied assumption in this ranking that those at the bottom, most of which are developing countries, should aspire to the level of quality of death of the highest ranked countries. We have argued against this homogenising tendency in end-of-life interventions and strategy.^47^ Moreover, recent evidence shows that even within the UK, ranked highest in the Quality of Death Index, there is much concern and a measure of disagreement about how well palliative and end-of-life care are being delivered ‘at scale’.^48 49^

We suggest that free-flowing compassion is still possible in the global South but that it too will soon experience similar constraints to those of the global North. Both North and South are proceeding towards an environment in which free-flowing compassion will be difficult to set in operation. The structural factors that inhibit compassion may prove far stronger than the religious, ethical and moral dimensions that promote it. At the moment, we cannot provide specific recommendations to address this problem, but we argue that unrealistic assumptions have been made about the role of compassion in end-of-life care and these idealist aspirations must be tempered by a more structural assessment of potential. Compassion may be hard to argue against, but without being tied to concrete action, it runs the risk of being or becoming a rather fragile set of exhortatory rhetorics.
